# Enhancing cuff removal accuracy in paediatric patients with long-term venous catheters: The role of ultrasound

**DOI:** 10.1177/11297298261448717

**Published:** 2026-05-20

**Authors:** Bjarte Toppe, Kristian Sønstabø, Tore Reikvam

**Affiliations:** 1 Haukeland University Hospital, Bergen, Norway; 2 Clinical Department 2, University of Bergen, Bergen, Norway

**Keywords:** Techniques and procedures, ultrasoundguided cuff localization, patient safety and procedural accuracy, paediatric long-term venous catheters, echogenicity of catheter cuff

## Abstract

Accurate localization of the cuff is crucial when performing a surgical cutdown procedure for the removal of long-term venous catheters. This small quality improvement study evaluated the effectiveness of ultrasound as a method for precise cuff localization in paediatric patients. Twelve children undergoing catheter removal were assessed by physicians using ultrasound in addition to traditional palpation to determine cuff position. In all cases, the cuff location identified by ultrasound corresponded precisely with the actual position observed during the surgical cutdown procedure. In three cases where palpation alone did not allow the physician to confidently identify the cuff location, ultrasound proved to be particularly useful for accurate localization. These findings suggest that the cuff of long-term venous catheters exhibits high echogenicity, and that ultrasound might serve as a valuable adjunct to palpation, enabling more accurate cuff localization during surgical cutdown procedures.

## Introduction

Children requiring long-term intravenous treatments such as chemotherapy, parenteral nutrition or haemodialysis often have long-term venous access catheters placed. These catheters are tunnelled under the skin and feature a small polyester cuff attached near the entry site. The polyester cuff is typically positioned 1–3 cm from where the catheter enters the skin. After 3–6 weeks, the surrounding tissue of the cuff becomes fibrotic, anchoring the cuff securely to the soft tissue. This helps prevent catheter displacement and provides an additional layer of protection against infections.

After completing treatment, long-term tunnelled catheters with cuffs must be removed. A common approach is to simply pull the catheter without addressing the cuff, but this carries a significant risk of leaving the cuff subcutaneously, potentially leading to future complications.^1–4^ Catheter manufacturers recommend removal of the cuff together with the catheter, a practice that is also supported by several current clinical guidelines.^5,6^ To ensure this, many hospitals commonly perform a small transverse surgical incision near the cuff, carefully freeing it before removing the catheter. Currently, no studies have been conducted in children to compare which method is most effective. But there are reports of complication where retained catheter cuff have caused chronic inflammation. In addition, resistance encountered during catheter removal may create uncertainty for the physician as to whether the cuff remains firmly adherent to surrounding tissue or whether intravascular complications are present. The latter may represent a more serious situation, such as intravascular catheter retention, and therefore requires careful assessment.^7^

At our hospital, the preferred method is surgical cuff removal using a cutdown procedure. In most cases, the cuff can be readily palpated beneath the skin in children, allowing accurate localization of the incision site. However, in some children, cuff localization by palpation can be challenging. Accurate localization is therefore important to avoid unnecessarily large incisions. We conducted a quality assurance study at our hospital to assess whether ultrasound is a valuable adjunct for physicians in cuff identification compared with palpation alone in children.

## Materials and methods

Twelve paediatric patients scheduled for Hickman catheter removal underwent pre-procedural ultrasound examination to identify the location of the catheter cuff. At our institution, all catheter removals in children are performed under general anaesthesia. The procedures were performed by four physicians experienced in ultrasound-guided techniques, particularly vascular procedures, although they had limited prior experience with ultrasound for cuff localization. All ultrasound examinations were performed after induction of anaesthesia and were therefore not associated with patient discomfort. Ultrasound imaging was performed using a Venue system (GE HealthCare) and required approximately 1–2 min to complete, adding minimal time to the overall procedure. Most catheters removed were manufactured by Bard or Vygon, the standard catheter brands used at our institution; however, catheters from other manufacturers were also included when insertion had been performed at external hospitals. For each removal, the physician completed a form recording the number of days the catheter had been in place, the child’s age group (0–3 years, 4–10 years or 11–16 years), and the distance from the skin to the cuff as measured by ultrasound.

The physician also recorded whether the cuff was ‘very easy’, ‘easy’ or ‘difficult’ to palpate. Cuff visibility on ultrasound was graded as ‘very good’, ‘good’ or ‘poor’. Finally, the physician performed a subjective assessment of the agreement between the cuff position identified by ultrasound and its actual location during surgical removal. Agreement was categorized as ‘very good’, ‘good’ or ‘poor’.

Comparison of procedural outcomes, such as incision length or operative time before and after the introduction of ultrasound, was not possible, as these data had not been systematically recorded prior to implementation of ultrasound guidance. The physician was also asked to record any adverse events on the data collection form.

The study was a quality assessment of a standard method used at our hospital and received approval from the hospital’s research department.

## Results

Twelve children aged 0–18 years were included, with the majority (8 children) between 4 and 10 years of age. The duration of catheter placement ranged from 74 to 804 days, with a mean duration of 301 days. When palpation alone was used to locate the cuff, the physician rated the process as ‘poor’ in three cases, ‘very easy’ in five cases and ‘good’ in four cases. In contrast, cuff visibility on ultrasound was rated as ‘very good’ in nine cases and ‘good’ in three cases, with no examinations rated as ‘poor’. In all 12 cases, the physician reported that the cuff location identified by ultrasound corresponded very well with the actual cuff position observed during the cut-down procedure. No complications or adverse events were reported in any of the 12 cases.

## Discussion

The procedure for removal of long-term venous catheters has received less attention than catheter insertion. Crocoli et al.^8^ published a protocol in 2014 describing the removal of long-term central venous catheters in paediatric patients. This protocol provides a comprehensive and well-structured overview of catheter removal techniques. The authors recommended a surgical cut-down procedure for cuff removal; however, no guidance was provided on how to best achieve precise localization of the cuff prior to incision.

**Figure 1.**
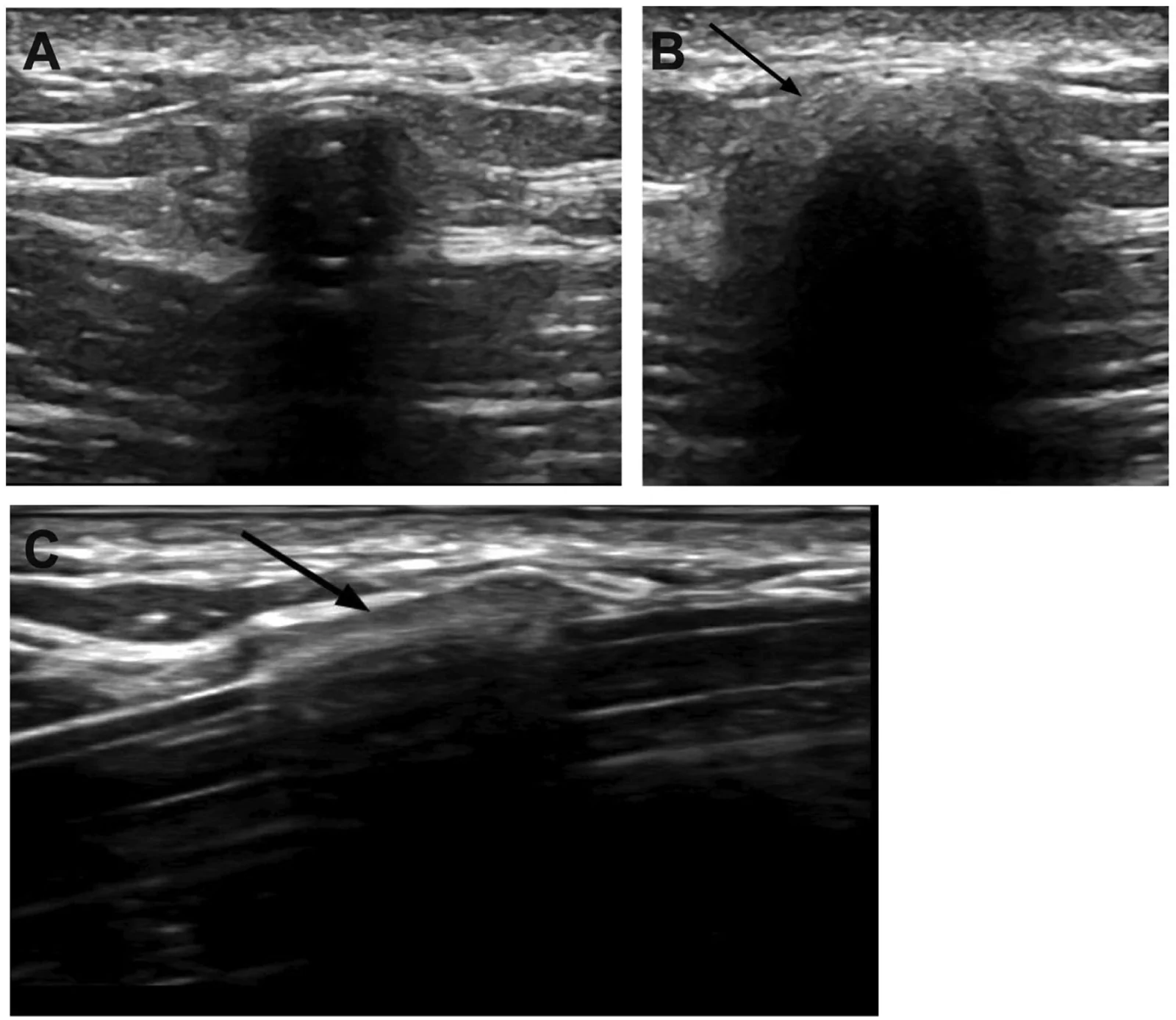
(a) Shows a transverse section of the catheter without a visible cuff, (b) A half-moon-shaped shadow over the catheter represents the cuff (indicated by the arrow) and (c) Presents a longitudinal section of the catheter, where the cuff appears as a thickening on the catheter (indicated by the arrow). Notably, the presence of the cuff reduces the echogenicity of the catheter wall.

Our findings suggest a potential role for ultrasound in the localization of long-term venous catheter cuffs in paediatric patients. The cuff consistently demonstrated high echogenicity (Figure 1), which likely contributed to the favourable visibility ratings observed; in most cases (*n* = 9), cuff visibility was assessed as ‘very good’, and no examinations were rated as ‘poor’. Although the sample size was limited, ultrasound-based localization corresponded with the actual cuff position in all 12 cases, supporting the feasibility of this technique.

The majority of catheters had been inserted at our institution, while a smaller number originated from other hospitals. No apparent differences in cuff echogenicity were observed between catheter origins; however, the sample size was insufficient to allow meaningful comparison. Overall, these findings indicate that ultrasound may serve as a useful adjunct in situations where cuff palpation is challenging, such as deep cuff placement or the presence of fibrotic tissue following infection.

Barnacle and Mitchell^9^ concluded in a 2005 study that ultrasound is an effective tool for cuff localization in adults. Our findings are consistent with these results and further suggest that ultrasound is also a reliable modality for locating the cuff in paediatric patients during removal of long-term venous catheters. In children, precise cuff localization is particularly important for surgical cut-down procedures, as smaller and more accurate incisions may improve cosmetic outcomes and potentially reduce the risk of complications such as bleeding and infection, although these complications are uncommon. However, the sample size in our study is insufficient to draw any conclusions regarding these potential benefits.

The discussion of whether the cuff should be removed or not is beyond the scope of this study. As previously mentioned, the preferred procedure at our hospital is surgical cut-down for cuff removal. However, there has been some discussion about initially attempting the traction method. If the cuff remains in situ – which has been reported to occur in approximately 60% of cases^10^ – it could then be located using ultrasound and subsequently removed. There are also reports indicating that a retained cuff, following catheter removal, is easily visible with ultrasound.^4^ However, this is not current practice, and future evaluation of this method would be required if implemented.

## Conclusion

Despite the small sample size, this local quality assurance and feasibility experience suggests that ultrasound may be useful for cuff localization during removal of long-term central venous catheters using a surgical cut-down technique, particularly in cases where cuff palpation is uncertain. Based on our clinical experience following the introduction of ultrasound guidance, the procedure appears to be time-efficient and may assist physicians in achieving a precise, minimal incision directly over the cuff. Further studies are warranted to evaluate whether a traction-based approach, combined with ultrasound-guided cuff localization in cases of retained cuffs and followed by ‘rescue’ cuff removal, could represent a feasible option in selected paediatric patients.
